# The incubation period of Buruli ulcer (*Mycobacterium ulcerans* infection) in Victoria, Australia – Remains similar despite changing geographic distribution of disease

**DOI:** 10.1371/journal.pntd.0006323

**Published:** 2018-03-19

**Authors:** Michael J. Loftus, Jason A. Trubiano, Ee Laine Tay, Caroline J. Lavender, Maria Globan, Janet A. M. Fyfe, Paul D. R. Johnson

**Affiliations:** 1 Department of Infectious Diseases, Austin Health, Heidelberg, Victoria, Australia; 2 Victorian Department of Health and Human Services, Melbourne, Victoria, Australia; 3 Department of Medicine, Melbourne University, Parkville, Victoria, Australia; 4 Victorian Infectious Diseases Reference Laboratory, North Melbourne, Victoria, Australia; University of Tennessee, UNITED STATES

## Abstract

**Background:**

Buruli ulcer (BU) is a geographically-restricted infection caused by *Mycobacterium ulcerans*; contact with an endemic region is the primary risk factor for disease acquisition. Globally, efforts to estimate the incubation period of BU are often hindered as most patients reside permanently in endemic areas. However, in the south-eastern Australian state of Victoria, a significant proportion of people who acquire BU are visitors to endemic regions. During a sustained outbreak of BU on the Bellarine peninsula we estimated a mean incubation period of 4.5 months. Since then cases on the Bellarine peninsula have declined but a new endemic area has developed centred on the Mornington peninsula.

**Method:**

Retrospective review of 443 cases of BU notified in Victoria between 2013 and 2016. Telephone interviews were performed to identify all cases with a single visit to an endemic region, or multiple visits within a one month period. The incubation period was defined as the time between exposure to an endemic region and symptom onset. Data were subsequently combined with those from our earlier study incorporating cases from 2002 to 2012.

**Results:**

Among the 20 new cases identified in short-term visitors, the mean incubation period was 143 days (4.8 months), very similar to the previous estimate of 135 days (4.5 months). This was despite the predominant exposure location shifting from the Bellarine peninsula to the Mornington peninsula. We found no association between incubation period and age, sex, location of exposure, duration of exposure to an endemic region or location of BU lesion.

**Conclusions:**

Our study confirms the mean incubation period of BU in Victoria to be between 4 and 5 months. This knowledge can guide clinicians and suggests that the mode of transmission of BU is similar in different geographic regions in Victoria.

## Author summary

Buruli ulcer (BU) is a destructive skin and soft tissue infection caused by *Mycobacterium ulcerans*. Contact with an endemic area is the key risk factor for disease acquisition. Incubation period cannot be estimated in long-term residents of endemic areas but in Victoria, Australia a proportion of our cases are short term visitors. We previously estimated a mean incubation period of 4.5 months during a sustained outbreak on the Bellarine peninsula. Since then there has been a significant increase in Victorian cases, a decline in cases on the Bellarine peninsula and the rapid expansion of a new endemic area on the Mornington peninsula. We performed a new retrospective review of 443 BU notifications between 2013 and 2016, identifying a further 20 cases with single or tightly clustered (within 1 month) exposures to BU-endemic areas. We estimated a mean incubation period in the new group of 4.8 months, very similar to our previous estimate. This knowledge assists clinicians assessing patients with compatible symptoms and a distant exposure to an endemic area, and public health practitioners seeking to prevent transmission. Our finding also suggests that the mode of transmission of BU may be similar in different geographic regions in Victoria.

## Introduction

Buruli ulcer (BU) is a geographically-restricted skin and soft-tissue infection caused by *Mycobacterium ulcerans*, a toxin-producing environmental pathogen.

Classified by the World Health Organization (WHO) as a neglected tropical disease, BU is most prevalent in sub-Saharan Africa where it is a significant public health concern [1]. Cases have been reported in over 30 countries from regions including Central and South America, Southeast Asia, China and the Western Pacific [2]. The causative organism *M*. *ulcerans* was first definitively described in Australia in 1948 [3] where Buruli ulcer is also known as ‘Bairnsdale ulcer’ or ‘Daintree ulcer’ [4, 5].

Across the world the primary risk factor for BU is residence or travel within an endemic area. However within endemic areas the precise mode of transmission of BU remains incompletely understood. Recent research from Victoria implicates possums (common arboreal marsupials) as environmental amplifiers and reservoirs, and biting insects as likely mechanical vectors [6–11].

Previous international estimates of BU’s incubation period (IP) have varied significantly between a few weeks [12, 13] to five months or more [14]. However, the ability to estimate the IP of BU is hindered by most cases occurring in permanent residents of endemic areas, making it impossible to determine the exact timing of acquisition.

Victoria provides a unique opportunity to calculate the IP of BU as the condition is notifiable, diagnosis by polymerase chain reaction (PCR) is rapid and centralised, and endemic areas are systematically mapped by members of the WHO Collaborating Centre in Melbourne and Victorian Department of Health and Human Services (DHHS). A study from Victoria published in 2013 –based on interviews with 23 cases with single exposures to endemic areas between 2002 and 2012 (predominantly the Bellarine peninsula)–estimated a mean IP of 135 days or 4.5 months [15].

Recently, there has been a significant rise in the number of BU cases in Victoria, associated with a shift in its geographic distribution [16]. This increase in cases provided an opportunity to further refine the IP estimate for BU, assess any trend in IP over time, increase the power of statistical analyses exploring relationships between IP and clinical characteristics, and also to consider the public health implications of this information.

## Methods

The methods for the original study to calculate the IP of BU have been previously described [15]. These were replicated in our study with a small number of modifications outlined below.

BU has been a notifiable condition in Victoria since 1 January 2004 with data systematically collected by the Department of Health and Human Services (DHHS). Enhanced surveillance has been performed since 1 January 2011 with follow up of all cases by public health officers at the time of notification. For this study all notifications between 2013 and 2016 were retrospectively reviewed. To optimise data completeness, interviews were performed if exposure information was missing, or if notification data indicated the case may have had either a single exposure to an endemic area or multiple exposures within a one month period.

**Fig 1:**
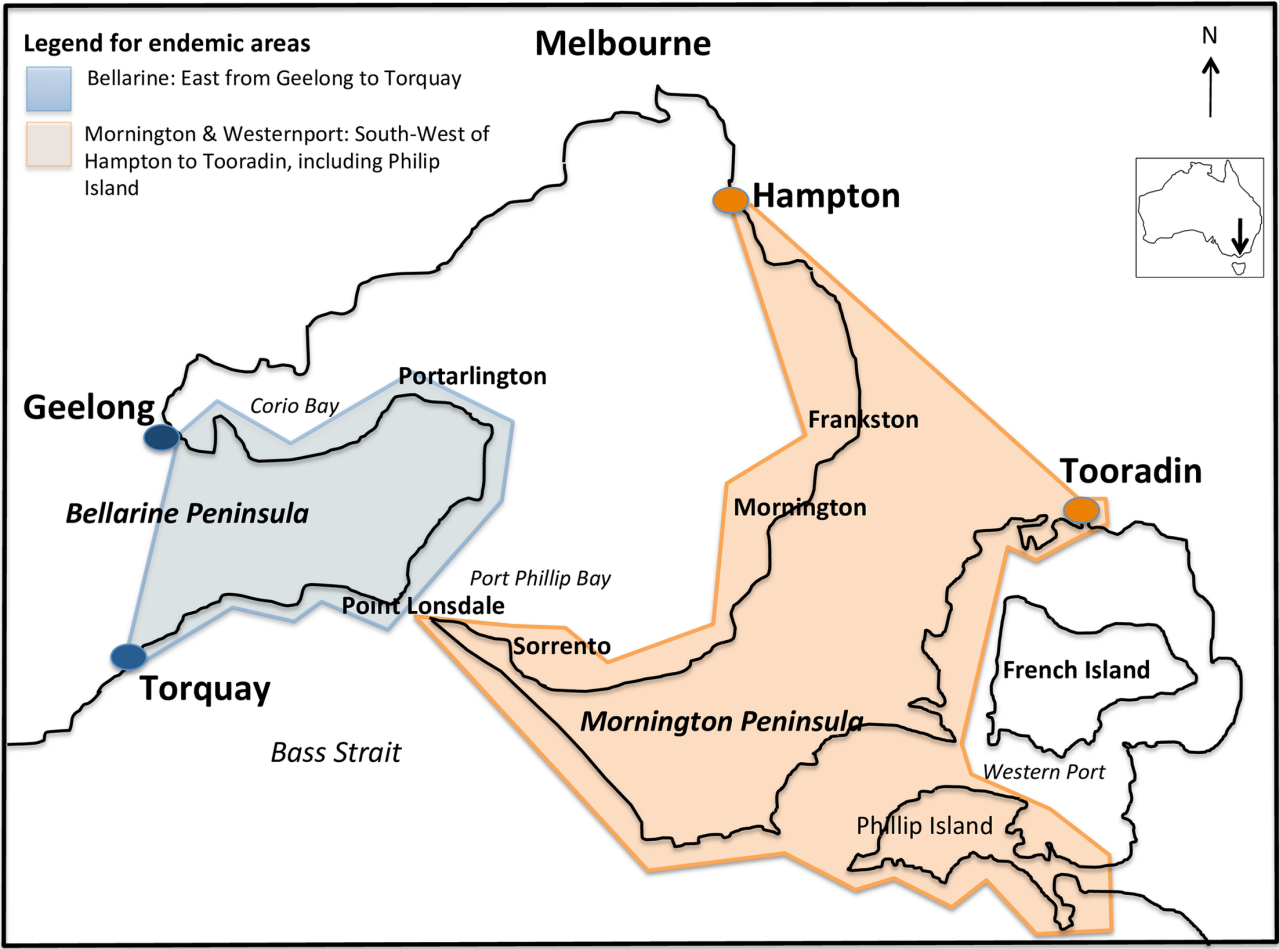
Regions near Melbourne considered endemic for *Mycobacterium ulcerans*. Image with adapted text from Trubiano et al. [15] as per the Creative Commons Attribution license. Small endemic region of East Gippsland not shown, 250km east of Melbourne.

The case definition for BU and the regions considered endemic remained unchanged from the previous study (Fig 1, S1 Table). The definition of the maximum period of significant exposure to an endemic area was shortened to one year (rather than two years) prior to symptom onset, based on the previously reported average IP of 4.5 months (range 1–9 months) [15].

Telephone interviews using a structured questionnaire were conducted with cases or next-of-kin in conjunction with review of the DHHS notification record, to obtain a detailed history of travel to endemic regions. The period of exposure was obtained (maximum one month) and a minimum and maximum IP value (IP range) for that patient calculated based on the time between their earliest (for maximum IP value) or latest (for minimum IP value) dates of exposure to an endemic area and their date of first symptom onset. The midpoint of the IP range was used to determine a point estimate of the IP for each case. Subsequently, mean and median IPs for the entire study cohort were calculated.

Other patient characteristics and data collected included age, sex, location of BU lesion, endemic area(s) visited, date(s) of exposure, date of first presentation to a healthcare worker, date of diagnosis of BU and treatment received.

These new data were subsequently combined with data from the original 2013 IP study to allow for comparison of trends over time and to increase the power of statistical analyses.

### Statistical analyses

Descriptive analyses were performed, with means or medians reported depending on the distribution of data. Mann-Whitney U tests or Kruskal-Wallis tests were used to explore any significant associations between IP (in days) and age, sex, endemic area visited, duration of exposure and site of lesion.

All data preparation and analysis was conducted using Microsoft Excel 2010 and Stata version 13.0 (Stata Corp., College Station, TX, USA).

### Ethics

All identifying data in this study were obtained under the legislative authority of the Public Health and Wellbeing Act 2008 and separate ethics approval was not required.

## Results

Of the 443 patients notified to DHHS with BU between 2013 and 2016, 177 (40.0%) had residential addresses outside endemic regions. Telephone interviews were conducted with 49/177 (27.7%) non-residents with missing exposure data or whose notification form could not exclude a single exposure period of less than one month. 20/49 (40.8%; 4.5% of the overall cohort) were confirmed to have only had a single visit to an endemic area, or multiple visits within a one month period; it was from these patients that the IP was estimated (S1 Fig).

The median age was 34 years (range 3–66), eight patients were male. The most common endemic region visited was the Mornington peninsula with 14 exposures followed by the Bellarine peninsula with 8 exposures. Two patients had single exposures on both the Mornington and Bellarine peninsulas within a one month period (Table 1).

**Table 1:** Characteristics of 43 cases from Trubiano et al. [15] and current study cohorts.

Characteristic	Previous cohort (n = 23)	Current cohort (n = 20)	Overall (n = 43)
**Age**–median (range)	28 years (6–76)	34 years (3–66)	32 years (3–76)
0–9	4 (17.4%)	2 (10.0%)	6 (14.0%)
10–17	4 (17.4%)	2 (10.0%)	6 (14%.0)
18–30	4 (17.4%)	5 (25.0%)	9 (20.9%)
31–60	6 (26.1%)	8 (40.0%)	14 (32.6%)
>60	5 (21.7%)	3 (15.0%)	8 (18.6%)
**Notification date**			
2003–2006	6 (26.1%)	NA	6 (14.0%)
2007–2009	4 (17.4%)	NA	4 (9.9%)
2010–2012	13 (56.5%)	NA	13 (30.3%)
2013–2014	NA	7 (35.0%)	7 (16.3%)
2015–2016	NA	13 (65.0%)	13 (30.2%)
**Sex**			
Male	15 (65.2%)	8 (40.0%)	23 (53.4%)
Female	8 (34.8%)	12 (60.0%)	20 (46.5%)
**Endemic area visited**^a^			
Bellarine peninsula	14 (60.9%)	8 (40%)	22 (51%)
Mornington peninsula	3 (13.0%)	14 (70%)	17 (40%)
Other–Victorian	4 (17.4%)	0 (0%)	4 (9%)
Other–Interstate	2 (8.7%)	0 (0%)	2 (5%)
**Season of exposure**			
Summer	14 (60.9%)	17 (85.0%)	31 (72.1%)
Autumn	4 (17.4%)	3 (15.0%)	7 (16.3%)
Winter	3 (13.0%)	0 (0.0%)	3 (7.0%)
Spring	2 (8.7%)	0 (0.0%)	2 (4.7%)
**Duration of exposure**			
Single day	4 (17.4%)	2 (10.0%)	6 (14.0%)
2–7 days	6 (26.1%)	8 (40.0%)	14 (32.6%)
8–14 days	2 (8.7%)	4 (20.0%)	6 (14.0%)
>14 days	11 (47.8%)	6 (30.0%)	17 (39.5%)
**Location of lesion**			
Leg–below knee	14 (60.9%)	13 (65.0%)	27 (62.8%)
Leg–knee or above	4 (17.4%)	1 (5.0%)	5 (11.6%)
Upper limb	5 (21.7%)	6 (30.0%)	11 (25.6%)
**Median days from symptom onset to diagnosis** (range)	71 (34–204)	112 (32–251)	85 (32–251)
**Median days from medical attention to diagnosis** (range)	35 (15–150)	48 (2–244)	43 (2–244)
**Mean duration of IP point estimate** (IP range)	135 days (32–264) 4.5 months (1–9)	143 days (61–277) 4.8 months (2–9)	138 (32–277) 4.6 months (1–9)

^a^ Cases (n = 2) that visited two endemic areas within a one month period are counted twice.

NA–Not applicable

Most exposures (n = 17) in our cohort occurred during summer, with the remainder in autumn. Half of the cases reported visiting an endemic area for one week or less; two cases visited only for a single day.

Over two-thirds (n = 14) of BU lesions occurred on the lower limbs, with the majority of these (n = 13) occurring below the knee. All remaining lesions were on the upper limbs.

The median time from symptom onset to diagnosis of BU in our cohort was 112 days (range: 32–251 days). The median time from first presentation to a healthcare worker to diagnosis of BU was 48 days (range: 2–244 days).

**Fig 2:**
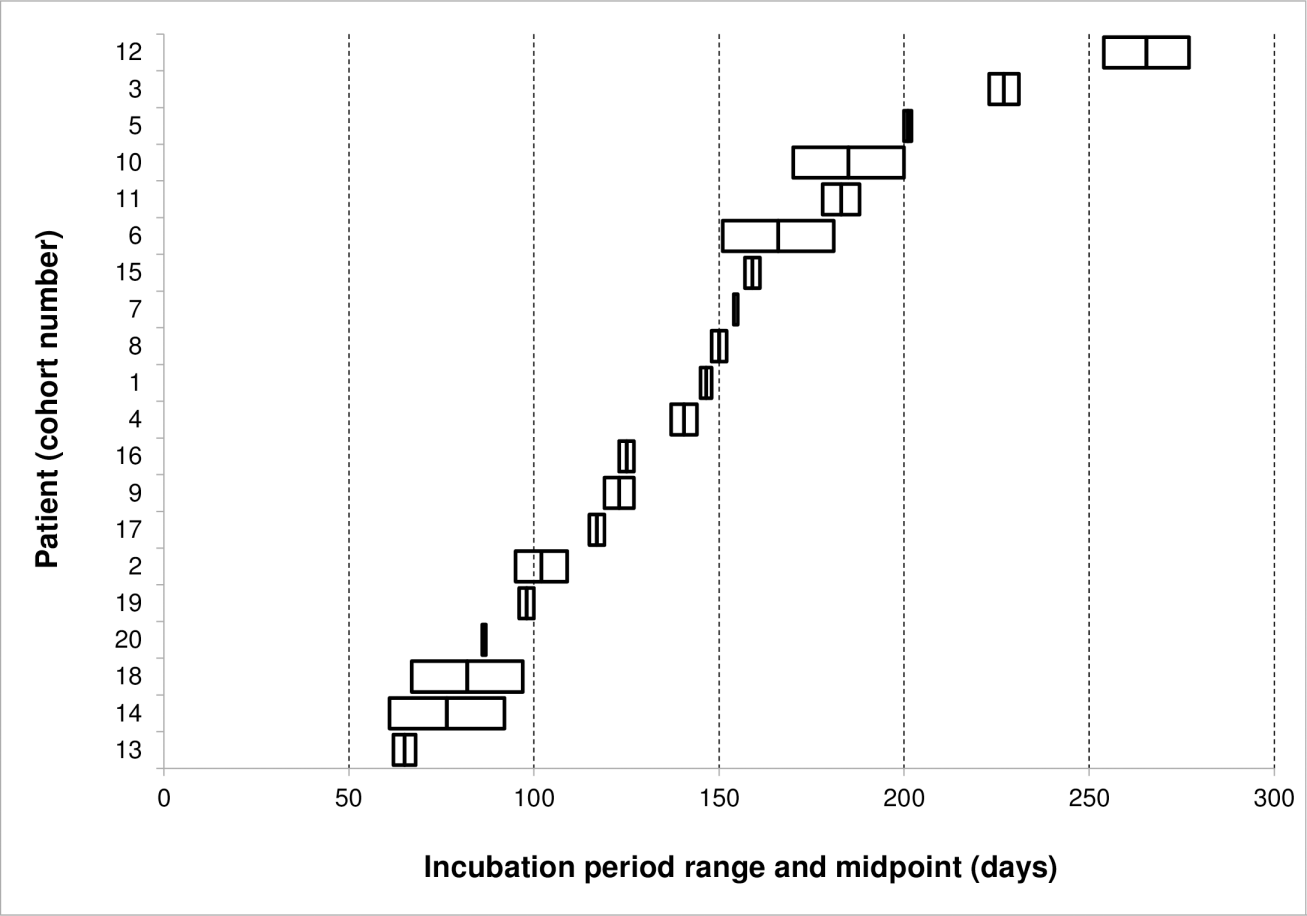
Incubation period ranges and midpoints for 20 patients in current study cohort.

The midpoint of the IP range was used to calculate a point estimate of the IP for each case. The mean and median of the IP point estimates were both 143 days, corresponding to 4.8 months (IQR 101–171 days, 95% CI 119.6–165.6 days) (Fig 2). The shortest IP recorded was 61 days (2 months), and the longest was 277 days (9 months).

In order to increase the study’s power and the likelihood of identifying any significant relationships between IP and demographic or clinical factors, the data from this study were combined with data from the previous Victorian study assessing the IP of BU (Table 1).

After combining datasets, the overall mean IP was 138 days and overall median IP was 136 days (IQR 107–162 days, 95% CI 123–153 days). Univariate analysis of the combined datasets did not identify any statistically significant relationships between incubation period and age (≤30 years vs. >30 years), sex, endemic area visited (Mornington peninsula vs. Bellarine peninsula vs. other), duration of exposure (≤7 days vs. >7 days) or location of lesion (lower limb vs. upper limb) (Table 2).

**Table 2:** Average incubation period in combined cohorts (n = 43) according to variables investigated.

Variable	N	Incubation Period		P value
		Mean (d/m)	Range (m)	
**Age**				0.11
0–30 years	21	128/4	2–8	
>30 years	22	148/5	1–9	
**Sex**				0.63
Male	23	140/5	1–9	
Female	20	136/5	2–9	
**Endemic area visited**^**a**^				0.46
Bellarine peninsula	20	151/5	2–9	
Mornington peninsula	15	139/5	1–9	
Other	6	122/4	3–5	
**Duration of exposure**				0.66
0–7 days	20	133/4	1–9	
>7 days	23	143/5	2–9	
**Location of lesion**				0.06
Lower limb	32	146/5	1–9	
Upper limb	11	115/4	2–5	

^a^ Cases (n = 2) that visited two endemic areas within a one month period were excluded from this analysis.

d–days. m–months.

## Discussion

Reliably estimating the IP of BU has been challenging due to an inability to define the exact timing of acquisition, as most sufferers of BU worldwide live permanently in endemic areas. However, Victoria’s unique epidemiology and comprehensive surveillance, combined with rising case numbers, allowed us to identify a group of patients for whom the incubation period could be accurately defined. A previous Victorian study on which ours was modelled reported an incubation period of 135 days (range: 32–264, IQR: 109–160, 95% CI: 113.9–156) [15]. Our study identified a similar number of new patients and reached a very similar estimate of 143 days, differing by only 6%.

Since the previous study there has been a large increase in the number of BU cases in Victoria, from an annual average of 30 cases between 2002 and 2007, to 50 cases between 2008 and 2012, and then to 111 cases between 2013 and 2016 (S1 Appendix). There has also been a pronounced shift in predominant exposure locations from the Bellarine peninsula to the Mornington peninsula [16]. This trend was reflected in the two studies, with only 3/23 in Trubiano et al. having a Mornington peninsula exposure, versus 14/20 in our study. Despite this change in geographic location–which could be accompanied by changes in environmental, host or vector factors–the IP of *M*. *ulcerans* infection appears the same in both regions.

Even after analysing all Victorian cases from the last 15 years for whom the IP could be accurately defined, we were unable to demonstrate a relationship between IP and demographic or clinical factors. It appeared that younger cases (≤30 years) and cases with upper limb lesions had shorter IPs, yet neither of these associations reached statistical significance (p values of 0.11 and 0.06 respectively). This may be due to insufficient power to detect an association due to small sample size, however could also represent the absence of any true relationship. Future studies incorporating further patients with single exposures would help clarify this issue.

Delayed diagnosis of BU is more common among non-residents of endemic areas [17], and the prolonged IP of BU is a likely contributor. Patients’ symptoms may appear months after their most recent travel to an endemic area, and both they and their local general practitioner (GP) are less likely to be aware of a rare mycobacterial infection restricted to specific coastal regions of Victoria. The median duration of symptoms prior to diagnosis of 112 days in our cohort is longer than the original IP study (71 days) and much longer than a recent case series of BU among residents and visitors to the Bellarine peninsula (42 days) [18]. However, *M*. *ulcerans* has been established on the Bellarine peninsula for well over a decade [19], and this region has received targeted physician-led GP education as well as public information campaigns from local councils that have not yet been replicated in the newly endemic areas of the Mornington peninsula. Long delays to the diagnosis of BU contribute to more advanced lesions and potentially worse outcomes. Analysis of the financial cost of BU has demonstrated an almost three-fold difference in treatment costs between minor and major infections [20].

Knowing the IP of BU allows for targeted public health actions. The Mornington and Bellarine peninsulas are popular beachside holiday destinations that receive over a million visitors during the summer months [21]. This period is when the greatest number of individuals are at risk of becoming infected with BU, and would be an appropriate time for strategies to reduce the risk of disease acquisition, such as vector control interventions by local councils or educational campaigns promoting avoidance of biting insects [10]. In contrast, the peak time for symptom onset–especially for non-residents of endemic areas–would be expected to occur 4–5 months later, during autumn and winter. This represents the most appropriate time for strategies to promote early diagnosis of BU through education and awareness-raising activities directed at both medical professionals and the general public.

It is only possible to study the incubation period of BU when the disease occurs in short term visitors who otherwise live outside BU endemic areas. The ratio of visitors to permanent residents in Victoria is almost 1:1 which is much higher than observed in Africa where BU is almost exclusively a disease of residents of endemic areas. Our study was based on recent cases who live in or visit two key endemic areas in Victoria: the Bellarine and Mornington peninsulas. All *M*. *ulcerans* isolates from Australia belong to the virulent “classical lineage” and are closely related to strains from Africa and Papua New Guinea [22]. A recent phylogenetic study of 178 Australian strains of *M*. *ulcerans* has shown that while the Bellarine outbreak is based on a single clone there are two distinct lineages causing human disease on the Mornington peninsula, particularly since 2012 [23]. We have not yet sequenced all isolates from all patients with MU in Victoria and so are not able to analyse the patients in the current study by strain. However the fact that the estimated incubation period appears the same on both peninsulas suggests that strain differences do not strongly influence IP.

There are several limitations to our study. First, the small number (<5%) of BU patients with single exposure period limited our ability to assess associations with the IP. This was partly addressed by combining our data with the previous study from 2013, more than doubling the number of cases to be analysed, however despite this no statistically significant associations were found. Second, patient recall is a potential concern, however our shorter study period (4 years vs. 11 years in Trubiano et al.) meant that incorrect recall was less likely. In addition, the ability to cross-reference enhanced surveillance forms completed by clinicians at the time of diagnosis further increased the accuracy of our data. Finally, our IP estimate was obtained from Victorian cases only, and may not be generalizable to tropical Australia or other major endemic foci such as sub-Saharan Africa.

Our estimate of an incubation period of 4.8 months (range 2–9 months) for Buruli ulcer confirms a previous similar estimate derived from an earlier group of patients who mostly acquired their infections in a different geographic location. This suggests that the mode of disease transmission is similar in different Victorian endemic regions. This knowledge also has important implications for understanding the epidemiology of BU and planning public health interventions to reduce disease acquisition and promote early diagnosis.

## Supporting information

S1 Checklist STROBE checklist. (DOCX)

S1 Table Regions in eastern Australia defined as endemic for *Mycobacterium ulcerans*. (DOCX)

S1 Fig Flowchart of patients included in the study. (PDF)

S1 Appendix Number of Victorian cases of Buruli ulcer, 2002–2016. (XLSX)
