# Supplementary material for: The incubation period of Buruli ulcer (Mycobacterium ulcerans infection) in Victoria, Australia – Remains similar despite changing geographic distribution of disease
Source: PLoS Negl Trop Dis. 2018 Mar 19;12(3):e0006323. doi: 10.1371/journal.pntd.0006323 (PMC5875870; doi:10.1371/journal.pntd.0006323)
Supplement: S1 Table — (DOCX) [file pntd.0006323.s002.docx]

**S1 Table. Regions in eastern Australia defined as endemic for *Mycobacterium ulcerans***

| **State** | **Regions** |
| --- | --- |
| Victoria (VIC) | Melbourne Bayside, East Gippsland, Westernport, Bellarine peninsula, Mornington peninsula |
| Northern Queensland (NQLD) | Daintree locality, Daintree River and adjacent coastal lowlands, Mossman Central coast |
| New South Wales (NSW) | Southern coastal regions |
