# Supplementary figures and images for: The incubation period of Buruli ulcer (Mycobacterium ulcerans infection) in Victoria, Australia – Remains similar despite changing geographic distribution of disease
Source: PLoS Negl Trop Dis. 2018 Mar 19;12(3):e0006323. doi: 10.1371/journal.pntd.0006323 (PMC5875870; doi:10.1371/journal.pntd.0006323)

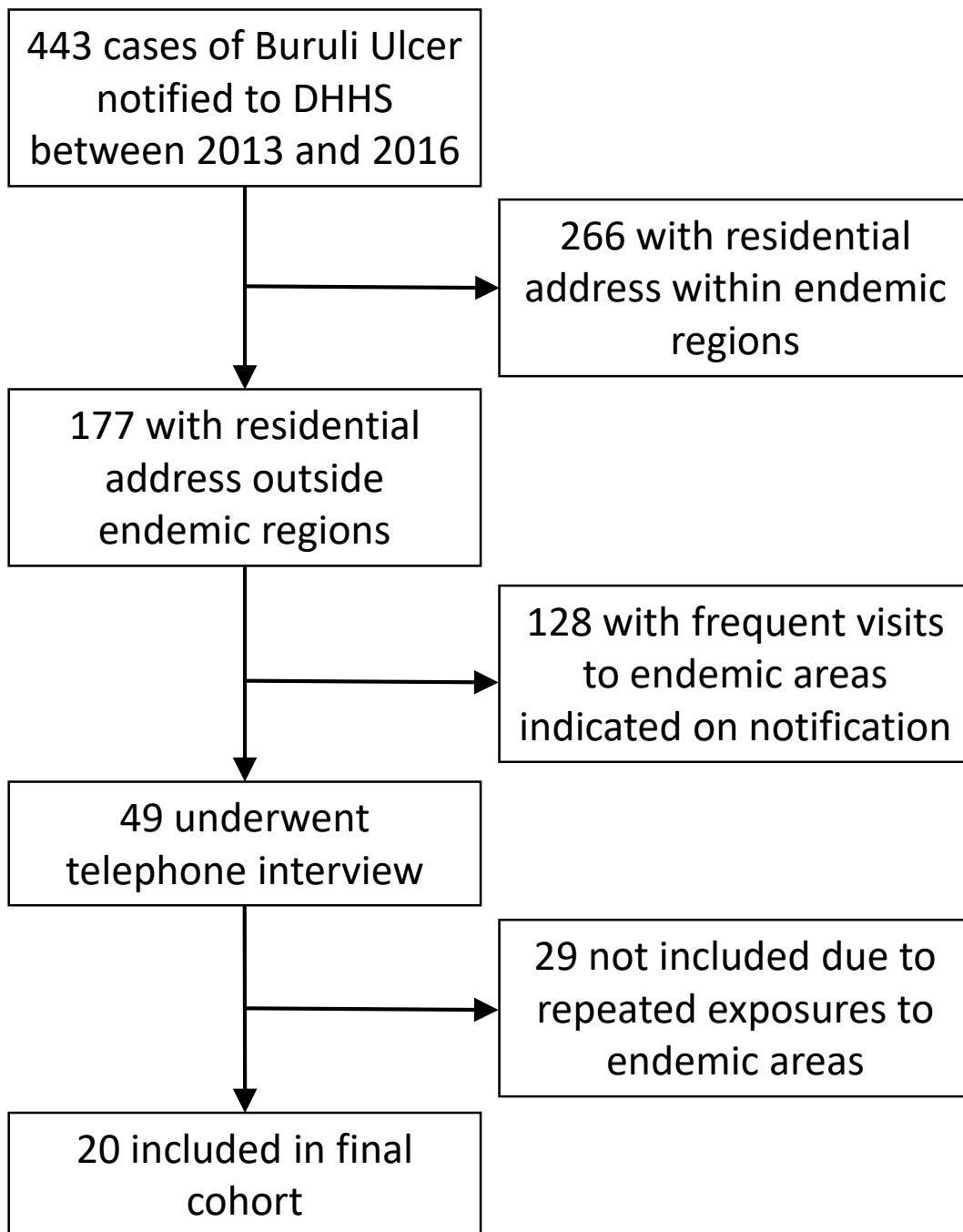

Supplement: S1 Fig — (PDF) [file pntd.0006323.s003.pdf]
